# Hydatid cyst disease of the lung as an unusual cause of massive hemoptysis: a case report

**DOI:** 10.1186/1752-1947-3-21

**Published:** 2009-01-23

**Authors:** Celal Tekinbas, Suleyman Turedi, Abdulkadir Gunduz, M Muharrem Erol

**Affiliations:** 1 Faculty of Medicine, Department of Thoracic Surgery, Karadeniz Technical University School of Medicine, 61080 Trabzon, Turkey; 2 Faculty of Medicine, Department of Emergency Medicine, Karadeniz Technical University School of Medicine, 61080 Trabzon, Turkey

## Abstract

**Introduction:**

Echinococcosis and/or hydatidosis is one of the most important zoonotic diseases in the world. In Turkey, echinococcosis is an endemic disease, however, hydatid disease of the lung is uncommon and usually caused by *Echinococcus granulosus*.

**Case presentation:**

In this report we describe a 17-year-old male patient who presented with massive hemoptysis due to hydatid disease of the lung.

**Conclusion:**

Although it is one of the less common causes of massive hemoptysis, hydatid disease of the lung requires greater attention in countries, such as Turkey, in which hydatid cyst disease is common.

## Introduction

Echinococcosis and/or hydatidosis is one of the most important zoonotic diseases in the world. In Turkey, echinococcosis is an endemic disease and the annual incidence of hydatid disease is 4.9 cases per 100,000 inhabitants [1]. However, hydatid disease of the lung is uncommon and usually caused by *Echinococcus granulosus*. In its adult stage, the parasite lives in the intestinal tract of carnivores such as dogs and cats, as well as in herbivores such as sheep. The head is composed of a double crown of hook-like structures, and the body is formed by three or four rings, the last of which bears the eggs. After being eliminated with feces, the eggs contaminate fields, irrigated land and wells. Herbivores ingest the eggs, which develop into larvae, or hydatids, within the viscera of these animals. The cycle is completed with the ingestion of the infected viscera by carnivores.

Humans contract the disease from water or food or by direct contact with dogs. Once the eggs reach the stomach, the hexacanth embryos are released. These pass through the intestinal wall and reach the tributary veins of the liver where they undergo a vesicular transformation and develop into hydatids. If they overcome the hepatic obstacle, they may become lodged in the lung, where they also transform into hydatids. If they advance beyond the lung, they may remain in any organ to which they are carried by the bloodstream. It has been shown that the embryos can reach the lung via the lymphatic vessels, bypassing the liver, and there is also evidence that the disease can be contracted through the bronchi [2].

## Case presentation

**Figure 1:**
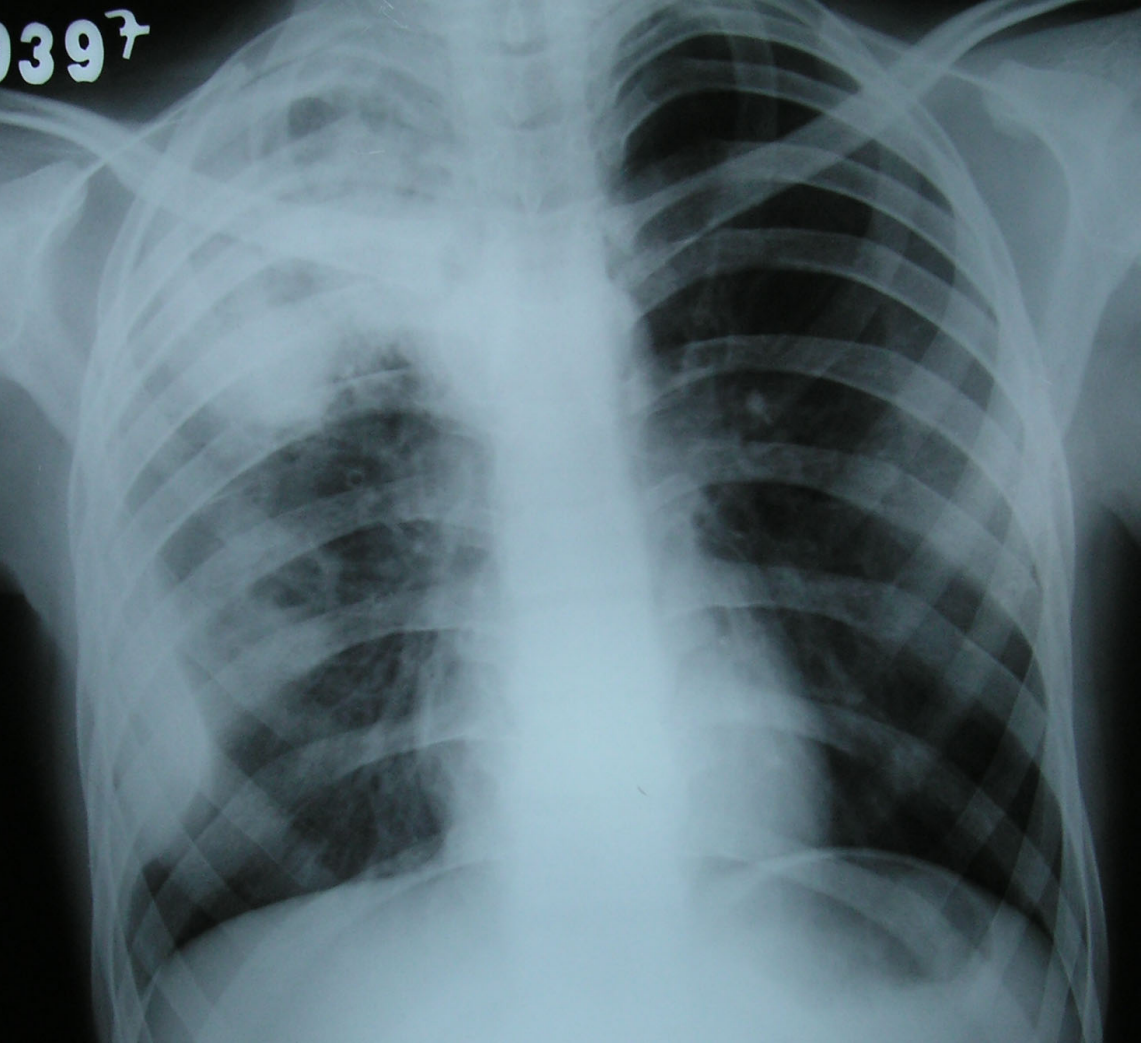
**A 5 × 6 cm circular lesion located in the apex of the right lung at X-ray**.

**Figure 2:**
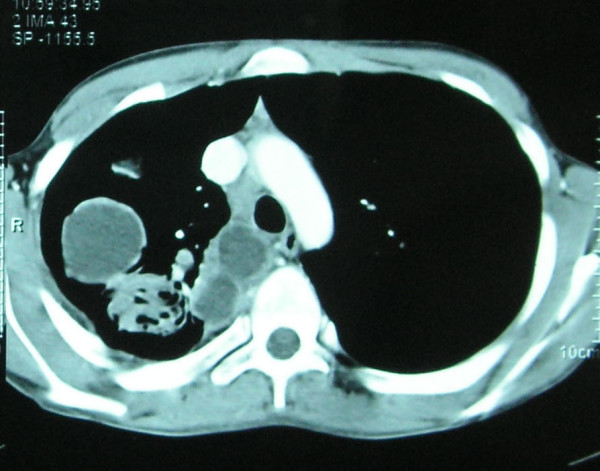
**A cystic lesion 4 cm in diameter revealed by computed tomography in the posterior segment of the upper lobe of the right lung and multiple lesions neighboring the former, some of which were cystic and others solid (largest was 2 cm in diameter)**.

A 17-year-old man, suffering from a cough, fever and weight loss for the previous 5 days, was admitted to the emergency department following hemoptysis a day before admission. He was conscious and pale. Blood pressure was 100/60 mmHg, pulse 110 beats per minute, respiratory rate 26 breaths per minute and body temperature 37.8°C. At physical examination, breathing sounds were roughened and inspiratory crackles were present in the right hemithorax. The other results of the physical examination were normal. (No other pathology was obtained.) In laboratory findings, values for C-reactive protein of 1.99 mg/dl, alanine transaminase of 26 U/litre, aspartate transferase of 14 U/litre, hemoglobin (Hb) of 9.7 mg/dl, Htc of 29.2%, mean corpuscular volume of 86 fl, prothrombin time of 29.7 seconds, activated partial thromboplastin time of 13.8 seconds and international normalized ratio of 1.09 seconds were obtained. A 5 × 6 cm circular lesion was located in the apex of the right lung at X-ray (Figure 1). A computed tomography (CT) scan revealed a cystic lesion 4 cm in diameter in the posterior segment of the upper lobe of the right lung and multiple lesions neighboring the former, some of which were cystic and the others solid (the largest was 2 cm in diameter). In addition, a 2 cm lesion was revealed in the superior segment of the lower lobe of the right lung (Figure 2).

On the first day of hospitalization, massive hemoptysis persisted. Increased respiratory failure and decreased Hb values (from 9.7 to 7.4 mg/dl) forced us to administer blood transfusion, after which surgery was indicated. The upper lobe bronchus of the right lung was completely obliterated while the anterior and posterior segments of the upper lobe were destroyed. In addition, one of the cysts in the lower lobe was ruptured. During surgery, lobectomy of the upper lobe of the right lung and cystectomy with capitonnage of the lower lobe cysts was performed. Capitonnage is obliteration of the pericystic cavity. The material obtained revealed hydatid cyst disease. Following medication with albendazole, the patient was discharged in a healthy condition on the eighth day postoperatively.

## Discussion

Hemoptysis in adults is most often caused by tuberculosis, bronchiectasis and trauma or bronchogenic carcinoma. Parasitic etiology is very rare. Small cysts are usually asymptomatic in hydatid disease. Coughing, chest pain and breathlessness are the common presenting symptoms. Hemoptysis as a presenting symptom is common in adult series, although massive hemoptysis is rare. The mechanism of hemoptysis may be due to pressure erosion of a bronchus or an obstructive effect with bronchial infection. There may be occasional rupture of cysts into the bronchus, resulting in massive hemoptysis. The underlying etiology for hemoptysis may be unknown in 20% of cases, but in cases with pulmonary hydatidosis, the clinical and radiological picture is so unique that it can be easily identified despite its rarity [3].

Diagnosis of an intact echinococcal cyst is usually based on a suspicion resulting from an unexpected finding on routine X-rays. Radiographically, the cyst appears as a homogeneous spherical opacity with definite edges. CT scanning and magnetic resonance imaging have added to the diagnosis of hydatid disease of the lung. Serological tests have limited diagnostic value. It is diagnosed by viewing the cystic membrane.

Hydatid cysts are typical, involving one lobe in 72% of cases, usually at the lung base [4-6]. In this case, multiple cysts were present in both lower and upper lobes of the right lung. The hydatid cyst not open to the pleura appears as a circular or oval image with well-defined limits, that can change according to its evolution. If the cyst ruptures, a radiological image of the pneumopericyst appears. If the content of the cyst is completely evacuated to the bronchial tree, a cavity similar to those observed in tuberculosis or pulmonary abscesses appears. However, if the content is only partially evacuated, a waterline image appears, commonly referred to as the Camelot sign [7]. Rupture of cysts may cause an anaphylactic reaction.

The conventional treatment of hydatid cysts in all organs is surgery. Medical treatment with albendazole is also effective in selected patients. Praziquantel may be added to albendazole. Surgical methods related to pulmonary cysts include cystotomy and enucleation of the intact cyst, with or without capitonnage, for complicated or intact cysts. The current treatment of hydatid disease of the lung is complete excision of the cyst, including the germinative membrane, with the maximum preservation of lung tissue [8]. Thoracotomy is the best procedure for removing a hydatid cyst, but video-assisted thoracic surgery is suggested for selected patients [9].

## Conclusion

This case report suggests that when a patient presents with massive hemoptysis, zoonotic infections, especially hydatid disease of the lung, should always be considered alongside other common causes of massive hemoptysis. Although it is one of the less common causes of massive hemoptysis, hydatid disease of the lung requires greater attention in countries, such as Turkey, in which hydatid cyst disease is common.

## Competing interests

The authors declare that they have no competing interests.

## Authors' contributions

CT was involved in the management of the patient as well as writing the case reports. ST took the photographs. AG and MME were involved in the correction of the manuscript as well as general supervision. All authors read and approved the final manuscript.

## Consent

Written informed consent was obtained from the patient for publication of this case report and any accompanying images. A copy of the written consent is available for review by the Editor-in-Chief of this journal.
